# Complete intracranial response to talimogene laherparepvec (T-Vec), pembrolizumab and whole brain radiotherapy in a patient with melanoma brain metastases refractory to dual checkpoint-inhibition

**DOI:** 10.1186/s40425-018-0338-6

**Published:** 2018-04-06

**Authors:** Zoë Blake, Douglas K. Marks, Robyn D. Gartrell, Thomas Hart, Patti Horton, Simon K. Cheng, Bret Taback, Basil A. Horst, Yvonne M. Saenger

**Affiliations:** 1 Columbia University Medical Center, Hematology/Oncology, 650 West 168th Street, New York, NY 10032 USA; 2 NewYork-Prebsyterian/Columbia, Hematology/Oncology, 177 Fort Washington Avenue, New York, NY 10032 USA; 3 NewYork-Prebsyterian/Columbia, Hematology/Oncology, 161 Fort Washington Ave, New York, NY 10032 USA; 4 NewYork-Prebsyterian/Columbia, Radiation Oncology, 161 Fort Washington Ave, New York, NY 10032 USA; 5 NewYork-Prebsyterian/Columbia, Surgery, 161 Fort Washington Ave, New York, NY 10032 USA; 6 NewYork-Prebsyterian/Columbia, Dermatopathology, 630 W 168th Street, New York, NY 10032 USA

**Keywords:** Melanoma, Brain metastases, Talimogene laherparepvec, T-Vec, Checkpoint inhibitors, Anti-PD1, Anti-CTLA4, Nivolumab, Ipilimumab, Pembrolizumab

## Abstract

**Background:**

Immunotherapy, in particular checkpoint blockade, has changed the clinical landscape of metastatic melanoma. Nonetheless, the majority of patients will either be primary refractory or progress over follow up. Management of patients progressing on first-line immunotherapy remains challenging. Expanded treatment options with combination immunotherapy has demonstrated efficacy in patients previously unresponsive to single agent or alternative combination therapy.

**Case presentation:**

We describe the case of a patient with diffusely metastatic melanoma, including brain metastases, who, despite being treated with stereotactic radiosurgery and dual CTLA-4/PD-1 blockade (ipilimumab/nivolumab), developed systemic disease progression and innumerable brain metastases. This patient achieved a complete CNS response and partial systemic response with standard whole brain radiation therapy (WBRT) combined with Talimogene laherparepvec (T-Vec) and pembrolizumab.

**Conclusion:**

Patients who do not respond to one immunotherapy combination may respond during treatment with an alternate combination, even in the presence of multiple brain metastases. Biomarkers are needed to assist clinicians in evidence based clinical decision making after progression on first line immunotherapy to determine whether response can be achieved with second line immunotherapy.

## Background

Immunotherapy (IO) has changed the clinical landscape for patients diagnosed with metastatic melanoma, with checkpoint inhibition driving the dramatic improvement in clinical outcomes over the past 5 years. Ipilimumab, a monoclonal antibody that blocks cytotoxic T-lymphocyte associated protein 4 (CTLA-4), an inhibitor of early T-cell activation and proliferation, was a first in class agent approved in 2011 [1]. Following ipilimumab, programmed cell death protein 1 (PD-1) inhibitors, pembrolizumab and nivolumab, which reduce T-cell suppression at a later stage in immune response, were developed and demonstrated higher response rates (25–40% vs. 4–13.9%) when compared to chemotherapy, in addition to a dramatic improvement in overall survival (OS) (1-year OS 72.9% vs. 42.1%) [2, 3]. Following the success of these agents, numerous other immunotherapies have entered development and are currently at various stages in clinical trials with several agents recently approved.

Although these agents have prolonged the lives of many patients with metastatic melanoma, more than half of treated patients will not respond, and patients who initially respond remain at risk for progression [2, 3]. In an effort to increase the number of patients who may benefit from IO, as well as enhance the depth of response in responders, combination immunotherapy approaches have emerged. Recently, dual CTLA-4/PD-1 inhibition with ipilimumab/nivolumab has been approved for melanoma based on favorable progression free survival (PFS) relative to nivolumab alone [4].

Talimogene laherparepvec (T-Vec), a first-in-class therapeutic cancer virus, was approved by the FDA for the treatment of advanced melanoma in October 2015. Derived from an attenuated strain of herpes simplex virus type 1 (HSV-1), the virus has been genetically modified to secrete granulocyte-macrophage colony-stimulating factor (GM-CSF) and is administered by direct injection into accessible melanoma lesions. In addition to inducing chemotaxis through GM-CSF, the virus is believed to potentiate a systemic anti-tumor immune response through exposure of tumor antigens following infection of neoplastic cells. T-Vec was approved based on phase III data showing that patients treated with T-Vec had a target lesion response rate of 64% and a systemic overall response rate of 26.4% vs. 5.7% in patients injected with GM-CSF subcutaneously [5]. For patients with either multiple soft tissue or visceral metastasis, reduction, defined as a decrease in tumor volume of greater than 50%, was seen in 34% of non-visceral lesions and 15% of visceral lesions [6]. Since its approval, a trial with combination T-Vec and ipilimumab has suggested synergy with checkpoint inhibition [7]. There is an ongoing trial of T-Vec with pembrolizumab, with interim analysis demonstrating promising response rates [8]. Of note, and of particular relevance to our patient, patients with brain metastasis (BM) were excluded from all of the above studies.

In melanoma, up to 60% of metastatic melanoma patients will be diagnosed with brain metastasis over follow up, and approximately a third of those patients will die from progression of their brain metastases [9]. Management of these patients is challenging in many solid malignancies, in large part due to most cytotoxic agents having poor CNS penetrance. Traditional chemotherapeutic agents have been particularly ineffective in management of patients with melanoma brain lesions, as these agents demonstrate poor activity even in the management of systemic disease. However, several studies indicate that IO may offer much needed treatment options for patients with melanoma metastatic to the brain.

Efficacy data for IO in melanoma brain metastasis is limited as historically these patients have been excluded from large clinical trials. In a study of IO in the treatment of brain metastases of various histologies, which included 18 patients with untreated melanoma brain disease, pembrolizumab demonstrated similar intracranial and extracranial disease control rates (42% vs. 50%), with 4 partial responses and 4 patients with stable disease (SD) on CNS imaging. After a median follow-up of 11.6 months, median OS had not been reached [10]. Dedicated melanoma phase II trials evaluating combination with pembrolizumab and nivolumab are currently underway. While data on combination therapy is particularly limited, in a small series, 9 patients with metastatic melanoma, 6 of which had brain metastasis, were treated with combination IO with nivolumab and low dose ipilimumab (1 mg/kg), and no increase in neurologic toxicities were observed [11]. Ipilimumab/nivolumab combination immunotherapy is currently being investigated in phase II setting (NCT02320058) as well as compared to monotherapy with nivolumab (NCT02374242), with promising preliminary data recently released [12–14].

While beyond the scope of this review, targeted therapy, with BRAF and MEK inhibitors, have demonstrated considerable activity with melanoma brain metastases [9]. These agent represent critical therapeutic option in patients with BRAF mutation. Unfortunately, as would be expected, as seen with systemic disease control, durable responses are rare [9]. As such, patients with melanoma even with brain metastases, are in need in new therapeutics.

## Case presentation

A 68 year-old male with a past medical history of a distant and previously excised melanoma of unknown depth on his back, presented in January 2016 with a new skin lesion on his right forearm. The lesion was 1.5 cm in diameter, and ulcerated. He was additionally noted to have a firm right axillary mass. A biopsy of the arm lesion was performed and was notable for an 8.7 mm deep ulcerated melanoma with 5 mitotic figures/mm^2^. PD-L1 and BRAFV600E were negative by immunohistochemistry (IHC). Next generation sequencing performed on tumor tissue demonstrated an NRAS 182A > T Q61L mutation. Fine needle aspiration (FNA) of the axillary node was simultaneously performed and confirmed regional melanoma metastasis. A positron emission tomography (PET) scan demonstrated diffuse distant metastasis, including hypermetabolic lesions in the left sacrum, ilium, sternum, and several hypermetabolic enlarged right axillary nodes, the largest of which measured 5.2 × 3.2 cm. A brain magnetic resonance imaging (MRI) revealed two small parenchymal lesions in the right frontal lobe with diameters of 7.7 and 4.3 mm and a right cerebellar lesion. The patient underwent GammaKnife radiosurgery to all detectable lesions and was started on combination ipilimumab/nivolumab.

**Fig. 1:**
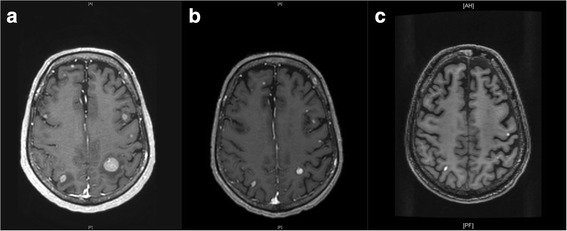
CNS response following radiotherapy and immunotherapy. **a** CNS lesions following gamma knife surgery (GKS), prior to whole brain radiation therapy (WBRT) and Talimogene laherparepvec (T-Vec). **b** Three months post WBRT and initiation of T-Vec (**c**) Six months following T-Vec

Unfortunately, following two doses of combined ipilimumab/nivolumab, CNS imaging revealed numerous new brain metastases, bilaterally. The lesion in the left anterior parietal lobe had increased from 7x9x7 mm to 12x15x14 mm (Fig. 1a). In late April, whole brain radiation therapy (WBRT) was recommended, which included 3750 cGy in 15 daily fractions. Checkpoint inhibition was initially continued despite CNS progression on MRI, as the patient had softening of his nodal metastases on upper extremity, which was felt to potentially represent systemic response, but was subsequently discontinued following his fourth cycle as he had progression of disease on clinical exam with increase in size of his lymphadenopathy.

At this point, systemic chemotherapy was discussed, but after the patient refused this option, a trial of pembrolizumab and T-Vec was administered. An initial dose of 4 mL (10^6^ plaque forming units (PFU)/mL) of T-Vec was injected into his dominant axillary mass that measured 7.5 cm in diameter. Two weeks after initiation of T-Vec, the patient received his first dose of pembrolizumab immediately following completion of WBRT. The patient continued receiving T-Vec every 2 weeks and pembrolizumab every 3 weeks with visible diminishment in size of injected lesions and the axillary lesion resolving to two separate lesions, each measuring approximately 2.5 cm.

**Fig. 2:**
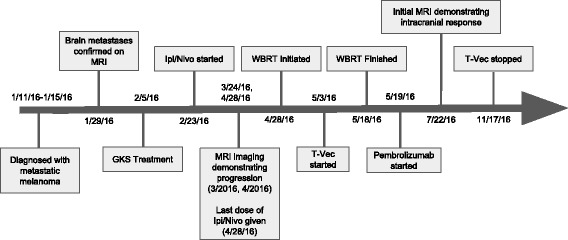
Timeline of the patient’s clinical course. GKS = Gamma knife surgery, WBRT = Whole brain radiotherapy. Ipi/Nivo = Concurrent ipilimumab and nivolumab

Notably, in late July, 11 weeks after initiating T-Vec, a brain MRI revealed a decrease in size of his metastatic lesions with associated decrease in surrounding vasogenic edema and no new lesions noted (Fig. 1b). PET/CT demonstrated a mixed response with improvement of disease in liver, bones and injected axilla, while there appeared to be an increase in other nodal sites. Over follow up, he had repeat PET scans which continued to demonstrate a mixed systemic response, however, his brain imaging demonstrated continued response to therapy (Fig. 1c). In total, he received 13 treatments of T-Vec over 6 months, prior to discontinuation for disease progression with development of new bone lesions and enlarging lymphadenopathy (Fig. 2). The patient did not experience any grade 3–4 toxicities while receiving T-Vec and pembrolizumab. His main complaint was significant fatigue and memory impairment, which was likely polyfactorial and due to WBRT, CNS metastasis, as well as related to IO. While on ipilimumab/nivolumab he developed a transient diarrheal illness which resolved without intervention and a rash which required topical corticosteroids. He died 16 months after initially being diagnosed with metastatic melanoma of innumerable brain metastases.

**Fig. 3:**
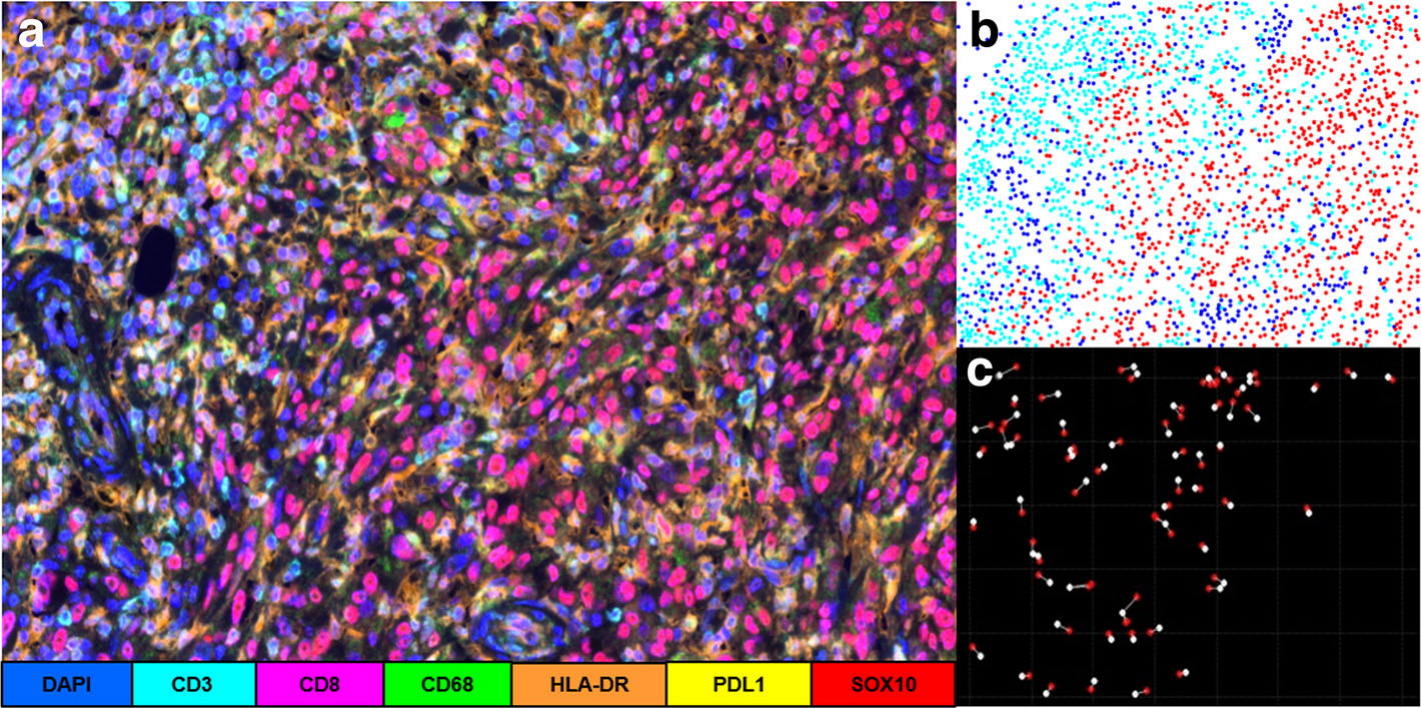
Representative tissue analysis using Quantitative Multiplex Immunofluorescence (qmIF). **a** 4 μm tissue section slide is processed using sequential qmIF protocol which uses tyrosine signal amplification following application of secondary antibody. After tissue processing, multispectral tissue images are obtained. **b** Cellular phenotyping is performed using companion software (inForm) which uses machine learning to perform automated cell phenotyping based on representative cell selection producing a Cartesian map with the X,Y coordinates of each cell in the imaged tissue, along with its assigned phenotype. **c** Spatial analysis can be performed using a variety of approaches including nearest neighbor calculation. As an example, distance between tumor cells (SOX10+) and nearest neighboring CTL (CD3 + CD8+) is being depicted

Of interest, our patient consented to enrollment on a bio-specimen collection protocol at our institution for the purposes of evaluating biomarkers predictive of response to IO in metastatic melanoma. As such, we performed quantitative multiplex immunofluorescence (qmIF) on his pre-treatment tumor tissue. QmIF allows for simultaneous (“multiplex”) evaluation of up to 7 antigens on the same full section tissue slide using proprietary secondary fluorophores (PerkinElmer) of distinct wavelengths that can be independently visualized after image capture using a multispectral camera (Fig. 3). This pathology evaluation platform has been validated for tissue evaluation in the research setting [15].

**Fig. 4:**
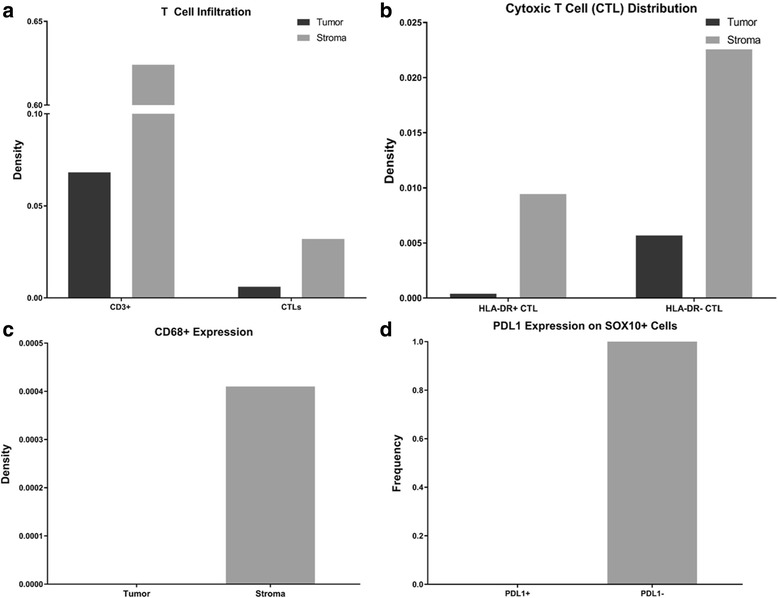
Cellular phenotyping of the tumor immune microenvironment (TME). **a** T Cell infiltration – Total CD3 vs. CTL infiltration in tumor (dark) and stroma (light) (**b**) Cytotoxic T cell (CTL) activation by tissue location and activation status, HLADR+ active/HLA-DR- inactive. **c** CD68 distribution by location. **d** SOX10+ PDL1 expression. Of note, representative tissue images did not include any PDL1+ CD68 cells or PDL1+ tumor (SOX10+) cells

QmIF analysis showed that within his primary tumor, our patient exhibited a high density of CD3 positive cells, approximately 62.4% of nucleated cells in the stroma, with 3.2% co-expressing CD8. PD-L1 expression on tumor cells was 0% on all evaluated fields. Macrophages (CD68+) comprised a minute subpopulation of immune cells and were exclusively found in the stroma (Fig. 4).

## Discussion

### Combination immunotherapy

Despite IO offering durable treatment options for many patients living with metastatic melanoma, nearly half of treated patients will not respond, even with combined checkpoint inhibition. T-Vec is distinct from other IO agents currently available, in part, as it is locally administered but still demonstrates the capacity to induce response in non-injected lesions through systemic immune activation. This antitumor effect is likely due to both the cytotoxic capacity of T-Vec, resulting in increased neoantigen exposure to host antigen presenting cells (APC) following tumor lysis, as well as its ability to modulate the tumor immune microenvironment (TME). In the phase II study of T-Vec in melanoma, post-treatment tumor biopsies were obtained, and treated patients were observed to have lower levels of immunosuppressive cell types including CD4 + FOXP3+ regulatory T-cells and myeloid derived suppressor cells (MDSCs) within the TME of injected lesions as compared to reference controls [5]. In two patients for whom biopsies of both injected and non-injected tumors were available, the non-injected lesions demonstrated a phenotype containing intermediate levels of these immunosuppressive subtypes as compared to biopsy data from untreated patients. These findings suggest that T-Vec may promote a more effective antitumor immune response.

However, while non-injected lesions do regress with T-Vec, the systemic response rates are significantly lower than those seen with PD-1 checkpoint inhibition. Modest response rates in visceral metastasis seen with T-Vec suggest that this agent may be most effective when used in combination with other IO agents, including checkpoint inhibitors, which can re-activate exhausted T-cells. Ipilimumab, as well as pembrolizumab, have both been evaluated in the phase I setting with T-Vec, with significantly higher response rates appreciated, particularly at non-target sites, than with T-Vec alone. When combined with ipilimumab, 52% of non-target index lesions demonstrated regression with an immune related response criteria (irRC) overall response rate of 50% (4 complete responses, 5 partial responses), which is dramatically higher than the approximately 10% response rate seen with ipilimumab alone [5]. While patients with brain metastasis were excluded from these studies, our patient’s response to T-Vec and pembrolizumab suggests that this combination can result in a systemic immune response capable of treating disease within the CNS.

### Management of melanoma brain metastasis with immunotherapy

As was the case for our patient, complications associated with CNS metastases is a leading cause of cancer related mortality in melanoma. Response data for melanoma brain metastasis with IO is limited as these patients were excluded from landmark IO clinical trials.

However, following the promising preliminary response data in the phase I setting, multiple dedicated trials that focus specifically on patients with melanoma brain metastasis are underway. These studies are designed to evaluate treatment efficacy on CNS related endpoints including intracranial response (ICR), functional status, and survival without neurologic decline, and will provide critical insight on the use of IO for asymptomatic and symptomatic brain metastasis [16]. Currently, a three-arm phase II trial, which includes two cohorts of patients with asymptomatic brain lesions treated with either ipilimumab/nivolumab for four doses followed by nivolumab biweekly (cohort A) or nivolumab monotherapy (cohort B), is underway. The study also includes a third cohort (cohort C) receiving nivolumab monotherapy with symptomatic or refractory brain metastasis or leptomeningeal disease, a subpopulation of patients that have never been included in earlier trials. The principal endpoint is ICR with secondary endpoints including extracranial response, overall response rate (ORR) and PFS/OS. Preliminary results initially reported at the ASCO 2017 Annual Meeting, and recently updated at the 2017 World Congress of Melanoma meeting, are promising with patients treated with ipilimumab/nivolumab achieving the highest ICR of 46%, double the 20% observed in cohort B. Cohort C had the lowest ICR (6%) [12, 13]. As would be expected, grade 3–4 toxicities were most common in Cohort A (43%), driven by ipilimumab, and similar to those seen in prior studies. Of note, neurological adverse events were rare (6%), occurring in only 4 patients, with one patient developing radionecrosis [12]. One seizure and two headaches were deemed to be related to treatment [12]. A similar ICR rate of 56%, was seen in patients treated with ipilimumab/nivolumab on CheckMate 204, also presented at the ASCO 2017 Annual Meeting [14].

The prognosis of melanoma brain metastases patients requiring WBRT is poor with 3 to 4 months survival. Melanoma is considered to a highly radioresistant tumor, and most of these patients have continued cranial progression. On our review of the literature we are unable to identify a published complete ICR to WBRT alone in the setting of multiple melanoma brain metastases [17–20]. However there is mounting evidence that the radiation response may be synergistic when used in combination with IO [21, 22]. Radiation, like oncolytic viral therapy, offers the possibility of increased tumor antigen presentation and can participate in IO-instigated in situ vaccination. Therefore, radiation could serve to augment an effective systemic immune response. Frequently referred to as the “abscopal effect,” tumor regression has been documented at non-irradiated sites and been attributed to an anti-tumor immune response [23]. However, unfortunately, this phenomenon has not been to-date reliably invoked in clinical management and, in fact, in some instances immune suppression has also been documented in the context of radiation therapy. These variable findings may be, in part, due to the total dose of radiation delivered or the dosing schedule [24–26]. Several studies are currently underway, which will hopefully provide additional insight into how radiation can be used to enhance the activity of IO (NCT02716948, NCT02858869).

### Predictive & prognostic biomarkers in melanoma

With new IO agents in development and the rise of combination IO, there is a need for accurate biomarkers to assist clinicians in determining which patients are most likely to respond to IO, as well as which patients would be expected to respond to alternative IO therapy at progression of disease. For ipilimumab, an increase in tumor-infiltrating lymphocytes (TILs) as well as a high genomic mutational load have been shown to be predictive of response to therapy [27, 28]. Certain specific mutations, including the NRAS mutation detected in our patient, also have been identified as associated with response to IO [29]. For anti-PD-1 therapy, TIL profiling, specifically with an increased density of CD8 T-cells, as well as high PD-L1 expression (≥50% by immunohistochemistry), can identify patients with the greatest probability of achieving a clinical response. However, a significant percentage (~ 12%) of patients with low PD-L1 expression (< 10%) will respond to treatment [30–32]. Overall, none of these biomarkers are sufficiently precise at determining which patients are likely to respond and more importantly, which IO agent might be expected to be most effective. As such, novel genomic and tissue based evaluation techniques are currently in development with the goal of addressing this need [33].

Among these approaches is qmIF, which can provide a higher resolution evaluation of the tumor immune microenvironment as compared to traditional IHC. We include this data as a representative example of the type of analysis that can be performed. Although this patient’s tumor showed a significant degree of TIL infiltration, described by the evaluating pathologist as varying from focally “non-brisk” to “brisk,” the population of CTL (CD3 + CD8+), which are known to be critical in anti-tumor response to programmed death-ligand 1 (PD-L1) therapy, were consistently low [28]. Furthermore, PD-L1 expression, on tumor cells, albiet of limited utility, was also absent. While clinically validated biomarkers are required, we speculate that despite having a low CTL density, the CD3 population (presumably comprised of CD4+ cells, including T regulatory cells) detected with qmIF, may have been suggestive of the potential fora response to immunotherapy. Of note, while our patient would not have been a candidate for MASTERKEY-26 trial due to symptomatic brain metastases, our findings are consistent with the corollary studies performed on tumor specimens obtained on this trial. In this phase Ib cohort (*n* = 21), response to combined T-Vec/pembrolizumab did not correlate with baseline CTL infiltration [34]. These findings, would suggest, that oncolytic viral therapy may be capable of augmenting the activity of anti-PD-1 therapy even in patients with a “non-favorable” TME.

In this case, qmIF was performed on this patient’s primary lesion prior to receiving any immunotherapy treatment, however this analysis can be performed on the TME of metastatic tumors, including the CNS, and may provide additional insight into immunoediting mechanisms unique to the CNS. Clinicopathologic studies indicate that the CNS is not an “immune-privileged” organ and multiple studies have demonstrated that CNS metastasis frequently resembles primary tumors with regards to immune infiltrate [35]. TIL infiltration, when seen in the CNS, similar to extracranial lesions, has demonstrated prognostic importance when found in the TME of melanoma brain lesions [36].

However, as new therapies are developed, access to the TME should not be assumed to be the same between extracranial and intracranial metastases as there are unique features of the TME in the CNS which warrant specific evaluation for their functional and prognostic implication. These include microglial cells which are known to be capable of antigen presentation and can express PD-L1, as well as the absence of high endothelial venules, which are postulated to enhance immune cell recruitment into the TME [9, 37].

## Conclusion

Our patient benefited significantly from combination immunotherapy with Pembrolizumab and T-Vec, a first in class viral IO therapy approved for the treatment of melanoma. Immunomodulating therapies such as radiation and combination IO can offer non-chemotherapeutic treatment options for patients who are either refractory to initial IO treatment or demonstrate progression of disease. Our patient was primary refractory to ipilimumab/nivolumab, thus treatment options were limited.

Our patient’s clinical response to T-Vec/pembrolizumab following WBRT underscores the benefit that combination IO and synergistic therapies can offer patients, even following progression on dual checkpoint inhibition. The CNS response observed also underscores the importance of including patients with melanoma brain metastasis in future clinical trials with novel IO agents as they are developed. Clinicopathologic research focusing on the TME of both CNS and non-CNS melanoma lesions can reveal the critical components of an effective anti-tumor immune response, as well as mechanisms of immune escape. Ultimately these features will permit identification of precise biomarkers and development of future IO agents for the treatment and management of patients who are refractory to currently available therapies.

## Abbreviations

APC: Antigen presenting cells
BM: Brain metastasis
CTLA-4: Cytotoxic T-lymphocyte associated protein 4
FNA: Fine needle aspiration
GM-CSF: Granulocyte-macrophage colony-stimulating factor
HSV-1: Herpes simplex virus type 1
ICR: Intracranial response
IHC: Immunohistochemistry
IO: Immunotherapy
irRC: Immune related response criteria
MDSCs: Myeloid derived suppressor cells
MRI: Magnetic resonance imaging
ORR: Overall response rate
OS: Overall survival
PD-1: Programmed cell death protein 1
PD-L1: Programmed death-ligand 1
PET: Positron emission tomography
PFS: Progression free survival
PFU: Plaque forming units
qmIF: Quantitative multiplex immunofluorescence
SD: Stable disease
TILs: Tumor-infiltrating lymphocytes
TME: Tumor immune microenvironment
T-Vec: Talimogene laherparepvec
WBRT: Whole brain radiation therapy
